# Validation of a semi-automatic method to measure total liver volumes in polycystic liver disease on computed tomography — high speed and accuracy

**DOI:** 10.1007/s00330-022-09346-6

**Published:** 2023-01-14

**Authors:** Sophie E. Aapkes, Thijs R. M. Barten, Walter Coudyzer, Joost P. H. Drenth, Ivo M. A. Geijselaers, Sterre A. M. ter Grote, Ron T. Gansevoort, Frederik Nevens, Maatje D. A. van Gastel

**Affiliations:** 1 Department Nephrology, University Medical Center Groningen, University of Groningen, Hanzeplein 1, 9700 RB Groningen, The Netherlands; 2 European Reference Center RARE-LIVER, Hamburg, Germany; 3 Department Gastroenterology and Hepatology, Radboud University Medical Center, Nijmegen, the Netherlands; 4 Department Radiology, Universiteitsziekenhuis Leuven, Leuven, Belgium; 5 Department Gastroenterology and Hepatology, Universiteitsziekenhuis Leuven, Leuven, Belgium

**Keywords:** Polycystic liver disease, Polycystic kidney disease, Tomography, X-ray computed, Organ size, Radiology

## Abstract

**Objectives:**

Polycystic liver disease (PLD) is characterized by growth of hepatic cysts, causing hepatomegaly. Disease severity is determined using total liver volume (TLV), which can be measured from computed tomography (CT). The gold standard is manual segmentation which is time-consuming and requires expert knowledge of the anatomy. This study aims to validate the commercially available semi-automatic MMWP (Multimodality Workplace) Volume tool for CT scans of PLD patients.

**Methods:**

We included adult patients with one (*n* = 60) or two (*n* = 46) abdominal CT scans. Semi-automatic contouring was compared with manual segmentation, using comparison of observed volumes (cross-sectional) and growth (longitudinal), correlation coefficients (CC), and Bland-Altman analyses with bias and precision, defined as the mean difference and SD from this difference. Inter- and intra-reader variability were assessed using coefficients of variation (CV) and we assessed the time to perform both procedures.

**Results:**

Median TLV was 5292.2 mL (IQR 3141.4–7862.2 mL) at baseline. Cross-sectional analysis showed high correlation and low bias and precision between both methods (CC 0.998, bias 1.62%, precision 2.75%). Absolute volumes were slightly higher for semi-automatic segmentation (manual 5292.2 (3141.4–7862.2) versus semi-automatic 5432.8 (3071.9–7960.2) mL, difference 2.7%, *p* < 0.001). Longitudinal analysis demonstrated that semi-automatic segmentation accurately measures liver growth (CC 0.908, bias 0.23%, precision 4.04%). Inter- and intra-reader variability were small (2.19% and 0.66%) and comparable to manual segmentation (1.21% and 0.63%) (*p* = 0.26 and *p* = 0.37). Semi-automatic segmentation was faster than manual tracing (19 min versus 50 min, *p* = 0.009).

**Conclusions:**

Semi-automatic liver segmentation is a fast and accurate method to determine TLV and liver growth in PLD patients.

**Key Points:**

• *Semi-automatic liver segmentation using the commercially available MMWP volume tool accurately determines total liver volume as well as liver growth over time in polycystic liver disease patients.*

• *This method is considerably faster than manual segmentation through the use of Hounsfield unit settings.*

• *We used a real-life CT set for the validation and showed that the semi-automatic tool measures accurately regardless of contrast used for the CT scan or not, presence of polycystic kidneys, liver volume, and previous invasive treatment for polycystic liver disease.*

**Supplementary Information:**

The online version contains supplementary material available at 10.1007/s00330-022-09346-6.

## Introduction

Polycystic liver disease (PLD) is characterized by growth of > 10 hepatic cysts, leading to hepatomegaly [1, 2]. PLD is caused by two inherited diseases: autosomal dominant polycystic liver disease (ADPLD) and autosomal dominant polycystic kidney disease (ADPKD). In ADPLD, cyst formation is restricted to the liver, while in ADPKD, all patients develop kidney cyst and liver cysts develop in the majority of patients [1, 3].

PLD can lead to various symptoms that ultimately impair their quality of life [4, 5]. Symptoms are caused by the enlarged liver, which leads to mechanical compression of adjacent structures. This results in PLD-related symptoms such as pain, loss of appetite, abdominal herniation, and sometimes a disturbed body image [1, 2, 6]. Total liver volume is directly associated with symptom severity [5–7].

Total liver volume (TLV) is an important parameter to define disease severity. Longitudinal TLV measurements play a pivotal role in evaluating disease progression and the response to therapies in clinical trials and clinical practice [8]. Liver function remains unaffected, even in severe cases and is not a relevant disease outcome [1, 2]. In one of the largest PLD studies, mean liver growth was 3.9% per year in untreated patients [9]. Therefore, precise TLV measurement, with a precision smaller than the mean annual liver growth, is important to determine treatment effects [9, 10].

The gold standard to measure TLV involves manual contouring of the liver boundaries on each slice, using dedicated software [11, 12]. TLV is calculated by combining the contoured area and slice spacing [11, 12]. This method is time-consuming and therefore expensive, and requires expert anatomical knowledge, and different observers may obtain dissimilar results from the same scan. Complex anatomical deformations and different tissue types present in PLD preclude the application of regular semi-automatic segmentation software. Therefore, there is an unmet clinical need for faster, easier, and accurate (semi-)automatic methods to measure TLV on CT [10].

The aim of this study was to show that semi-automatic liver segmentation, using Siemens Multimodality Workplace (MMWP) tool “Volume,” is reliable and faster than manual segmentation for both cross-sectional and longitudinal analysis, in a representative, real-life, dataset of PLD patients with a wide variety of CT scan protocols and liver sizes.

## Materials and methods

### Study population

In this cohort study, PLD patients were recruited from existing databases from observational studies in two tertiary referral centers in the Netherlands. Patients were included if they had PLD and two axial CT scans, at least 6 months apart, were available. All CT scans had been made in the framework of clinical care in our centers or referral centers, and had therefore been made according to different scan protocols with different phases of contrast. The only exclusion criteria were incomplete CT scans (e.g., missing slices) or incomplete livers on the CT scan. CT scan characteristics (low dose, blanco, or phase of contrast) were noted in the database. Baseline parameters on underlying disease (ADPLD or ADPKD), body height, and surgical interventions before and between the two CT scans were collected from study databases or patient records.

### Manual TLV measurements

Manual TLV measurements were performed by trained observers using Pinnacle 3 (Philips Radiation Oncology Systems) and Analyze version 9.0 (Analyze Direct, Inc.). Liver boundaries were contoured on each axial slice and TLV was subsequently calculated by multiplying the contoured area by the spacing between slices. Extrahepatic structures (e.g., gallbladder, portal vein, and caval vein) were excluded from the liver boundaries if visible. Before inclusion in the database, all liver segmentations were checked for completeness and all reported volumes were recalculated by another independent observer. All observers were blinded for time point, patient ID, and previous measured volumes for all measurements.

### Semi-automatic measurements using MMWP Volume (Siemens)

Semi-automatic TLV measurements were performed by one experienced observer (W.C.), using the Siemens MMWP tool called Volume version VE61B. This method is referred to as “semi-automatic measurements.”

Using this tool, the observer coarsely contoured the liver every three slices. The program interpolates all intermediate slices which were adjusted by the observer if necessary. The program excludes structures from the contoured regions if they fall outside predefined Hounsfield unit (HU) densities. For scans with contrast, irrespective of contrast phase, HU boundaries are set at −15 and 195; HU limits of −15 and 125 are used for scans without contrast. This ensures exclusion of extrahepatic structures including fat tissue and vascular structures. The program then calculates TLV by multiplying the spacing between slices with the areas of traced volumes, comparable to manual TLV measurements. The observer was blinded for time point and patient ID for all measurements.

### Additional measurements using Syngo.Via (Siemens)

We performed TLV measurements with the Siemens postprocessing application Syngo.Via VB50 in a randomly selected subgroup of ten patients. This technique lacks HU density options and resembles manual segmentation. However, it allows interpolation between contours reducing the number of slices that require manual contouring. The observer can manually correct the interpolated boundaries. Liver volume is calculated by the program by multiplying the area per slice with the spacing between slices.

### Inter- and intra-reader variation per technique

We determined the inter- and intra-reader variation in a randomly selected subgroup of ten CT scans from ten different patients (cross-sectional) to compare the reproducibility of each segmentation technique. Three readers measured these ten CT scans twice with manual segmentation and semi-automatic segmentation. Two readers measured the same ten CT scans twice with Syngo.Via. All readers were blinded to the previous measurements.

### Measuring time per technique

We recorded the time needed to perform semi-automatic segmentation, Syngo.Via, and manual segmentation measurements.

### Statistics

Baseline characteristics are reported as mean ± standard deviation (SD) for normally distributed parameters and as median (IQR) for non-normally distributed continuous parameters. Categorical variables are reported as *n* (%). Absolute TLV measurements were compared with the Wilcoxon signed-rank test. Using the Bland-Altman analyses, we further investigated the agreement of cross-sectional TLV measurements between manual and semi-automatic methods on the first CT scan of every patient. Mean TLV of manual and semi-automatic measurements was calculated, and the difference between this measurements divided by the mean TLV * 100 to calculate the percentage difference. The bias and precision represent the percentual mean and SD of this difference between both measurements. We performed a one-sample *t*-test to test for statistical differences between the segmentation methods.

For our secondary outcome, determination of agreement in longitudinal data, we compared manually determined liver growth with semi-automatically determined liver growth. Again, we used the Bland-Altman plots and tested the difference with a one-sample *t*-test. Liver growth was defined as the percentual growth between the first and second scans, regardless of time between the two scans. Difference in growth percentage was calculated by [semi-automatic growth (%)] − [manual growth (%)] and bias and precision parameters were calculated as the mean of this difference and the corresponding standard deviation. We performed sensitivity analyses in the following predefined subgroups: (1) CT scans with and without contrast, (2) patients with a height-adjusted TLV < 3200 mL and ≥ 3200 mL, (3) patients with and without a history of surgical interventions, and (4) patients with ADPKD versus ADPLD.

Inter- and intra-reader variability were investigated with coefficients of variance (CVs). The CV is the ratio of the SD to the mean. The inter-reader CV was calculated with values obtained by different readers who measured the same CT scan. The intra-reader CV was calculated for each reader separately after measuring the same CT scans twice. The intra-reader CVs of all readers were averaged to yield a mean intra-reader CV plus standard deviation. The Wilcoxon signed-rank tests were used to determine whether these CVs significantly differed between manual segmentation and semi-automatic or Syngo.Via measurements.

We performed time measurements and calculated the mean from different readers for every scan and every method. In addition, we performed the Bland-Altman analyses with bias and precision parameters that compare manual segmentation with semi-automatic or Syngo.Via measurements in the same ten CT scans.

### Ethical statement

All UMC Groningen patients included in this study participated in earlier studies in which they gave approval for the use of their data for future studies. All Radboudumc patients are part of the international PLD registry. Given the non-invasive nature of the data collection, formal ethic’s approval was waived by the Radboudumc Ethics Committee. Our study was conducted in accordance with the guidelines for Good Clinical Practice (GCP) and the Netherlands Code of Conduct for Research Integrity.

## Results

**Fig. 1:**
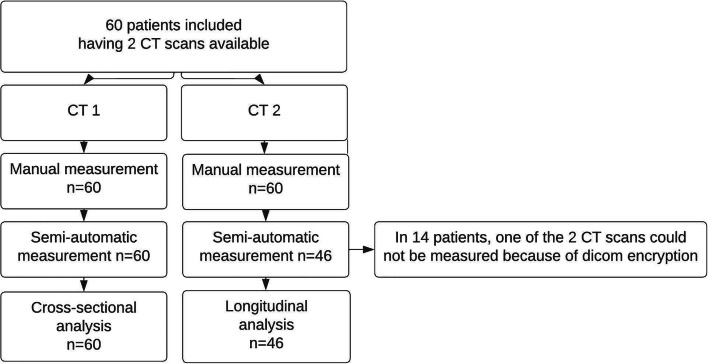
Flowchart of included patients and scans

**Fig. 2:**
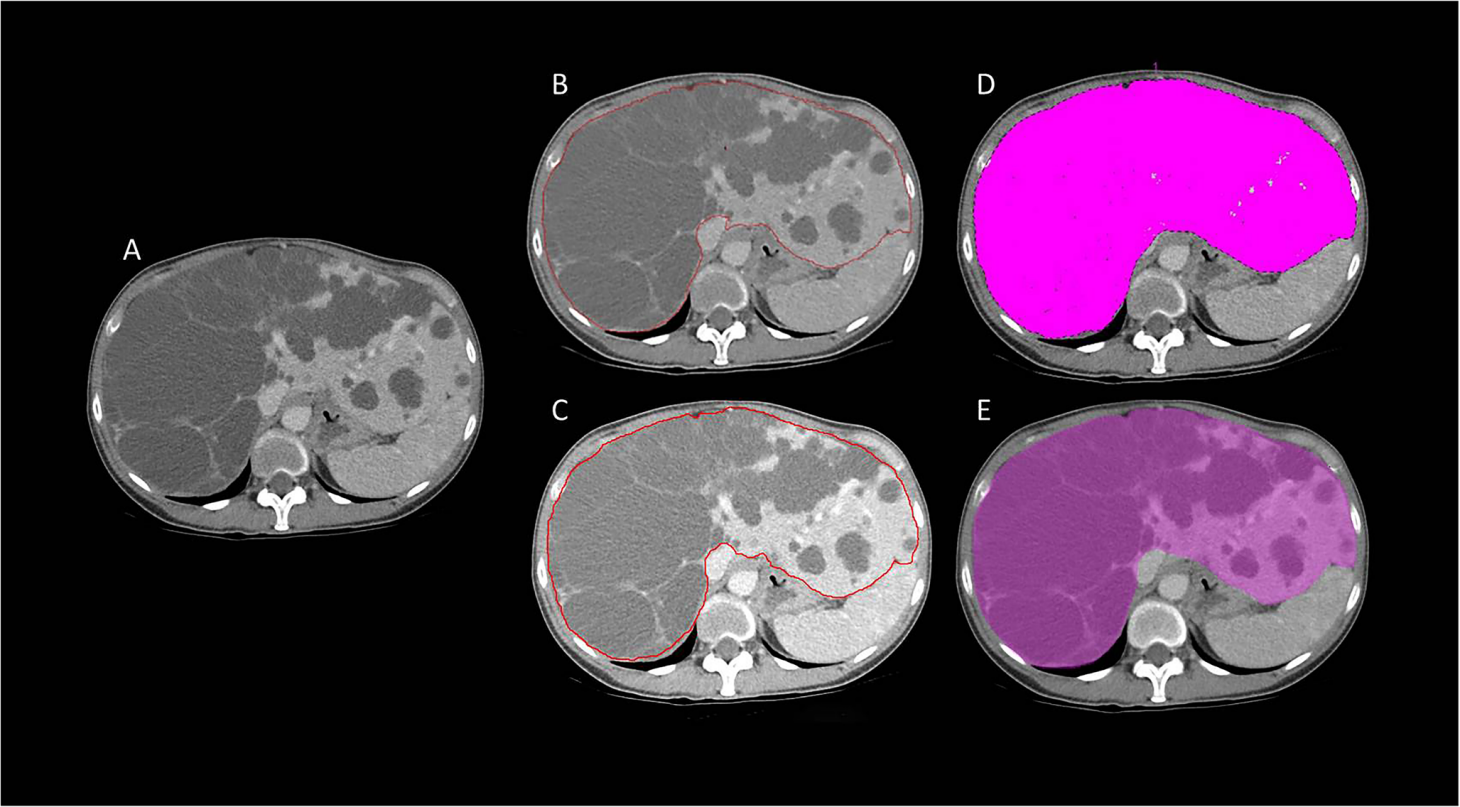
Image of the segmentation tools. **A** Unsegmented slice. **B** Manual segmentation with Analyze. **C** Manual segmentation with Pinnacle. **D** Semi-automatic segmentation. **E** Segmentation with Syngo.Via

A total of 60 PLD patients with two CT scans were included in the study. Volumetry was performed manually for all 120 CT scans. The semi-automatic method was not able to measure volumes on CT scans from the companies GE and Toshiba, due to encryption signatures present in these files. Consequently, we were not able to measure one of two CT scans in 14 patients. Therefore, cross-sectional measurements were obtained in all 60 patients and longitudinal data in 46 patients (Figs. 1 and 2).

### Patient characteristics

Baseline characteristics of the patients are shown in Table 1. The mean age of the patients included in the cross-sectional analysis is 51.2 ± 9.4 years (standard deviation (SD) 9.4) and 85% of patients were female. The majority of patients (58%) suffered from ADPKD, 38% suffered from ADPLD, and two patients (3%) had liver cysts but did not meet the criteria for PLD (> 10 hepatic cysts). Median TLV in the cross-sectional analysis was 5292.2 (3141.4–7862.2) mL and 18 patients (30%) received invasive treatment (e.g., aspiration sclerotherapy or cyst fenestration) for PLD before the first scan. In patients having ADPKD, mean creatinine was 106 (71–173) μmol/L. The subset of 46 patients having longitudinal data had comparable baseline characteristics.

**Table 1:** Baseline characteristics

	Cross-sectional *n* = 60	Longitudinal *n* = 46
Center		
Radboudumc	43 (71)	29 (63)
UMC Groningen	17 (28)	17 (37)
Sex		
Female	51 (85)	39 (85)
Male	9 (15)	7 (15)
Diagnosis		
ADPLD	23 (38)	17 (37)
ADPKD with PLD	35 (58)	27 (59)
ADPKD without PLD	2 (3)	2 (4)
Age years	51.2 (9.4)	51.7 (9.9)
Creatinine μmol/L*	106.0 (71.0–172.5)	110.0 (71.0–202.0)
TLV manual mL	5292.2 (3141.4–7862.2)	4854.1 (2624.9–8104.4)
CT with contrast	32 (53)	26 (57)
Venous contrast	22 (37)	
Late venous contrast	4 (7)	
Arterial contrast	6 (10)	
CT without contrast	28 (47)	20 (44)
Low-dose CT	19 (32)	
Blanco CT	9 (15)	
Invasive treatment PLD	18 (30)	16 (35)
Time between scans years		1.8 (0.8)

*Only for ADPKD patients. *ADPLD*, autosomal dominant polycystic liver disease; *ADPKD*, autosomal dominant polycystic kidney disease; *PLD*, polycystic liver disease; *TLV*, total liver volume; *CT*, computed tomography. Invasive treatment concerns cyst aspiration sclerotherapy or cyst fenestration

Data given as number (%), mean (SD), or median (IQR)

### Performance of the semi-automatic tool in cross-sectional TLV assessment

**Fig. 3:**
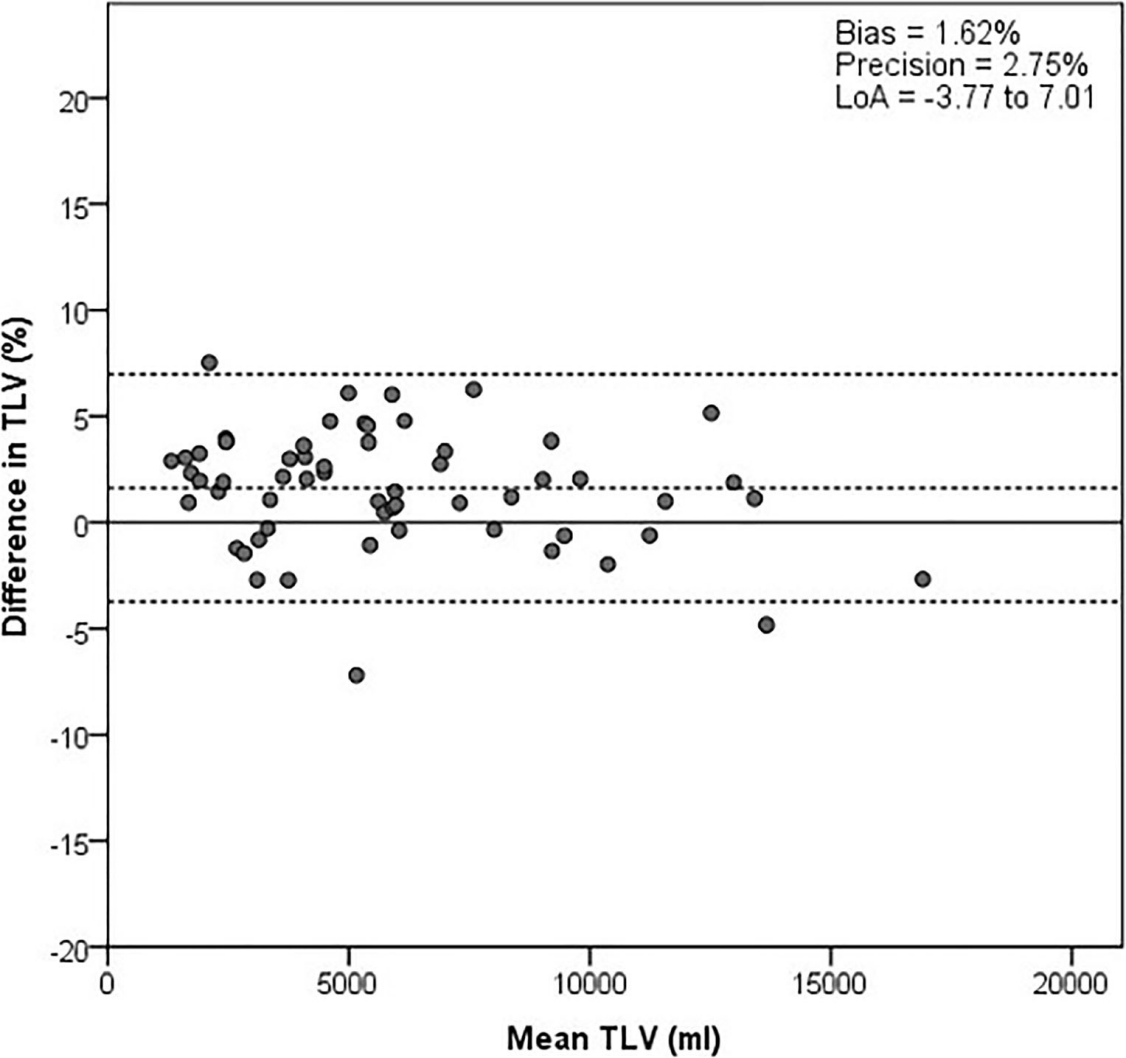
Bland-Altman plots of cross-sectional TLV measurement, compared between manual tracing and semi-automatic measurement. The solid line represents the reference and the dotted lines the bias and LoA. TLV, total liver volume; LoA, limits of agreement

Semi-automatic TLV measurements correlated with manual measurements (CC 0.998) but were slightly larger with a median of 5292.2 vs 5432.8 mL (*p* < 0.001, Table 2). This is also reflected in the bias of 1.62% and precision of 2.75% (*p* < 0.001, Fig. 3).

**Table 2:** Median total liver volumes using manual tracing and semi-automatic measurement. *TLV*, total liver volume; *CT*, computed tomography. TLV presented as median (IQR). Comparison of volumes was made with Wilcoxon signed-rank tests; growth was compared with paired *t*-tests

	*n*	Manual TLV	Semi-automatic TLV	*p*-value
Cross-sectional				
TLV CT 1 mL	60	5292.2 (3141.4–7862.2)	5432.8 (3071.9–7960.2)	< 0.001
Longitudinal				
TLV CT 1 mL	46	4854.1 (2624.9–8104.4)	5070.1 (2623.9–8108.9)	0.001
TLV CT 2 mL	46	4852.5 (2656.2–8444.7)	5147.0 (2745.8–8473.5)	0.005
Absolute growth mL	46	99.4 (789.8)	94.7 (855.7)	0.930
Percentage growth %	46	3.1 (14.0)	3.3 (15.5)	0.706

### Performance of the semi-automatic tool in detecting liver growth

**Fig. 4:**
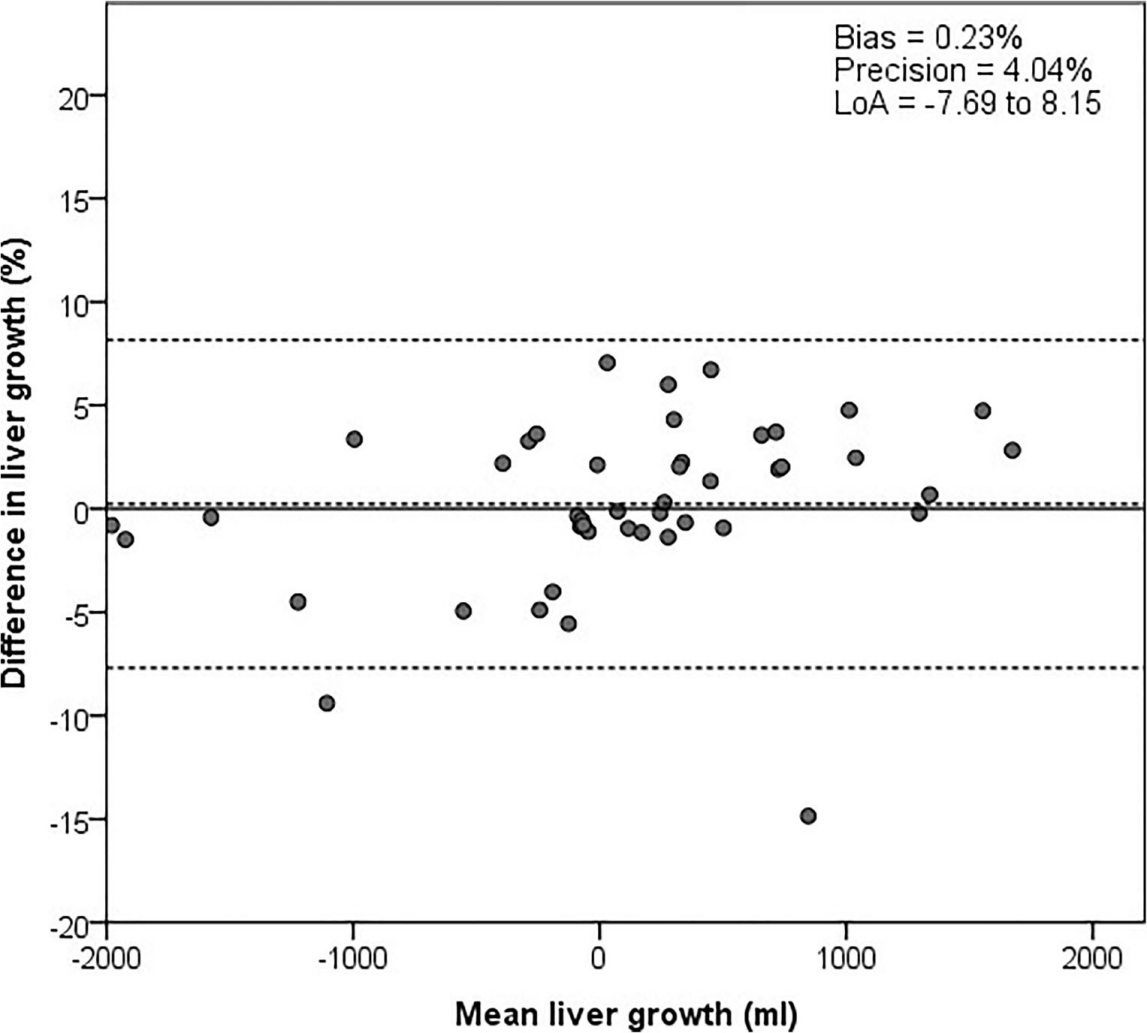
Bland-Altman plots of total liver growth between the first and second CT scans, compared between manual tracing and volume measurement. The solid line represents the reference and the dotted lines the bias and LoA. TLV, total liver volume; LoA, limits of agreement

While manual tracing consistently showed significantly smaller TLVs compared to semi-automatic measurements, no difference in liver growth was observed, for both absolute and percentage growth (*p* = 0.930 and *p* = 0.706, respectively, Table 2). Figure 4 shows a Bland-Altman plot for the longitudinal analysis of liver growth between the two CT scans, measured manually and semi-automatically. The bias and precision of total liver growth are 0.23% and 4.04%, respectively.

Figure 4 shows one outlier in the data: manual liver growth of 1701 mL and semi-automatic liver growth of −9 mL. Evaluation of the tracing showed that this was caused by a mistake in the second manual CT scan, where the right kidney was assigned to the liver (Supplemental Figure 1). If this case is excluded from the dataset, the absolute and percentage growth were not significantly different, with growth rates of 63.8 (± 760.5) mL and 2.9% (± 14.1) for manual tracing, and 97.0 (± 865.3) mL and 3.4% (± 15.6) for semi-automatic tracing (*p* = 0.381 and 0.271, respectively). The correlation coefficient would then be 0.960, with a bias and precision of 0.56% and 3.37%, respectively.

### Inter- and intra-reader variability and measuring time

In Table 3, the CVs for the inter- and intra-reader variability are given for manual tracing as well as the semi-automatic segmentation tool. The median segmentation time was 50 (46–78) min for manual segmentation and 19 (15–28) min for semi-automatic segmentation (*p* = 0.028, Table 3).

**Table 3:** Inter- and intra-reader variability for manual and semi-automatic measurements. Comparison for manual versus semi-automatic measurements was made using Wilcoxon signed-rank tests

	Manual	Semi-automatic	*p*-value manual vs semi-automatic
Inter-reader coefficient variation, %	1.21%	2.19%	0.260
Intra-reader coefficient variation, %	0.63%	0.66%	0.374
Measurement time, hour:minutes:seconds (IQR)	0:50:23 (0:46:42–1:18:15)	0:19:06 (0:15:00–0:28:29)	0.028

### Syngo.Via

A random subset of 10 CT scans was selected for measurement with Syngo.Via. In this subgroup, the accuracy of the Syngo.Via measurements was comparable to the semi-automatic segmentation method (Supplementary Table 1 and Fig. 2). The absolute TLV measurements of Syngo.Via were slightly larger than manual segmentations (median TLV 4265.8 versus 4124.8 mL, *p* = 0.007). Inter-reader CV was not significantly different from manual tracing, with values of 1.01% for Syngo.Via versus 1.21% for manual tracing (*p* = 0.445). The intra-reader CV was also comparable, being 0.54% for Syngo.Via, and 0.63% for manual segmentation (*p* = 0.333). Measuring time was not faster than manual tracing (48 (34–56) versus 50 min (46–78)) (*p* = 0.917), while being significantly slower compared to the semi-automatic measurement method of MMWP “Volume,” with a median measurement time of 19 min (15–28) (*p* = 0.05).

### Subgroup analysis

In the cross-sectional dataset, we compared the bias and precision of the semi-automatic tool in four predefined subgroups and found no differences. Results were comparable across CT scans with contrast (*n* = 32, bias 1.95%, precision 1.94%) and without (*n* = 28, bias 1.24%, precision 3.46%, *p* = 0.338). In addition, we compared performance of the semi-automatic program across different contrast phases, and between low-dose and blanco CT scans (Supplemental Figure 3). We observed no differences across contrast phases, but did observe a significant difference between low-dose and blanco CT scans (median percentage difference −0.3% and +3.8% respectively, *p* = 0.04, Kruskal-Wallis test).

Across patients with invasive PLD treatment before the scan (*n* = 18, bias 1.50%, precision 2.68%) and patients without invasive PLD treatment before the scan (*n* = 42, bias 1.67%, precision 2.81%, *p* = 0.830), results were comparable. We compared PLD patients with ADPKD (*n* = 37, bias 1.45%, precision 2.54%) with patients with ADPLD (*n* = 23, bias 1.89%, precision 3.10%, *p* = 0.549) and observed no differences. Lastly, we found that patients with manual height-adjusted TLVs of > 3200 mL/m (*n* = 31, bias 1.28%, precision 3.02%) were comparable to patients with manual height-adjusted TLVs ≤ 3200 mL/m (*n* = 27, bias 1.87%, precision 2.48%, *p* = 0.430). Two patients were excluded from this analysis since their height was missing.

## Discussion

We demonstrated that semi-automatic segmentation using the Siemens MMWP Volume tool accurately measures TLV (bias 1.62%, precision 2.75%) and liver growth (bias 0.23%, precision 4.04%) in a real-life dataset of patients with polycystic liver disease (PLD). Semi-automatic measurements were faster than manual measurements and were accurate regardless of contrast used in the CT scan, liver size, invasive interventions, and disease etiology. Only for low-dose CT scans compared to blanco CT scans, the performance was slightly different (median percentage difference −0.3% and +3.8% respectively, *p* = 0.04), which could probably be explained by the lower quality of the low-dose scans. We observed a minor but statistically significant difference in absolute TLV values between manual and semi-automatic measurements. This difference was consistent for baseline and follow-up scans leading to no difference in liver growth in both absolute (mL, *p* = 0.930) and percentage difference (*p* = 0.706). Thus, the semi-automatic method accurately measures liver volumes as well as growth, provided that the same method is used for consecutive measurements.

We determined the inter- and intra-reader variation in a subgroup of ten CT scans. The CVs were small and not significantly different for manual and semi-automatic segmentation. For semi-automatic segmentation, the intra-reader variability was 0.66%, showing that the same CT scan is reassessed accurately by the same reader. The inter-reader variability was slightly higher, but still good with a value of 2.19%, showing that the liver volume measurements were comparable between various readers. The inter-reader variability of manual tracing (1.21%) was comparable to the bias of semi-automatic measurements (1.62%), indicating that variation of these methods is quite similar.

In our study, we found a significant difference between observed volumes for the gold standard of manual tracing compared to semi-automatic measurements. However significantly different, this was a numerically small difference. Most importantly, there was no difference observed in the observed liver growth between the two methods. Liver growth is the most important parameter in PLD to assess disease progression. There was no difference in absolute as well as percentage difference in liver growth. Notably, liver growth should be assessed using the same method (either manual or semi-automatic volumetry), due to difference in absolute volumes between the methods.

Over the last years, several (semi-)automatic programs have been developed to measure TLV and/or total kidney volume in ADPKD and/or PLD [13–15]. The only fully automated program is currently only suitable for MRI [15]. This program has comparable bias (−1.6%) and precision (3.1%) to our semi-automatic TLV measurements and is currently used for studies and in clinical practice. CT scans come with several benefits over MRI as they are quicker, cheaper, and more widely available. Patients with contra-indications for MRI (e.g., claustrophobia or metal prosthesis) can also be scanned with CT. In addition, on MRI, artefacts can occur that do not occur on CT, especially in patients with large livers [16]. Lastly, abdominal CT scans are more often made for other purposes related to clinical care, and it would be beneficial if TLV can be measured on the same imaging modality. Therefore, (semi-)automatic volumetry programs are needed for CT.

Recently, Philips developed an automatic segmentation program with this purpose [14]. The bias of and precision (−1.1% and 4.0%) were comparable to our semi-automatic tool. However, the clinical value of this application has yet to be proven, since no longitudinal data on liver growth were shown in this study. In addition, it is unknown whether this program’s performance deteriorates when it is applied in real-life CT scans. Most programs require a standard scanning protocol, which is not always available in clinical practice.

The most important disadvantage of the developed semi- and fully-automatic programs [13–15] is feasibility of implementation in clinical practice. For their use, expert knowledge is required. We chose to validate a commercially available semi-automatic volumetry application, in order to simplify the implementation and transition to clinical practice. We chose the Siemens MMWP Volume tool as main outcome for this study. We observed that the semi-automatic volumes were comparable to the gold standard of manual tracing, while the MMWP Volume was significantly faster compared to both manual tracing and Syngo.Via (18, 50, and 48 min per scan, respectively).

However, since a newer version of this tool is available, we studied the performance of the newest Siemens volume tool “Syngo.Via” as well. We observed that this tool also measures slightly, but significantly larger TLV compared to manual tracing. The bias and precision for Syngo.Via TLV measurements were comparable to the bias and precision for MMWP measurement. Intra- and inter-reader variability were not significantly different compared to manual tracing. However, Syngo.Via TLV measurements were not faster than manual tracing (the gold standard), despite the option to use interpolation, which was lacking in our manual segmentation procedure. Probably, this could be explained because altering the contour or excluding structures in Syngo.Via was very time-consuming. In addition, every interpolated contour needed to be checked for correctness and adjusted if necessary. The MMWP Volume tool uses HU densities in volumetry, while this feature is no longer available in Syngo.Via. In our opinion, it is unfortunate that the HU density option is not available anymore in Syngo.Via, because this feature makes the measurements so much faster.

This study comes with a number of strengths. We used a set of real-life CT scans (with and without contrast and from different scanning companies) and manual contouring was performed by experienced readers. In addition, we used longitudinal data to validate the performance on liver growth. This is important, because liver growth is used to measure disease progression in clinical practice and is used as the main outcome in many clinical trials [8, 9, 17, 18]. We made a detailed assessment of inter- and intra-reader variability for all methods. This study also comes with limitations. First, the study has a limited sample size. However, the bias and precision presented in our current study are comparable to literature. Second, the manual measurements were performed by several readers, while a single reader performed all semi-automatic measurements. Since the inter-reader variation for manual segmentation was very small, we expect no effect on our study outcomes.

We demonstrated that the semi-automatic Siemens MMWP Volume tool is reliable, with comparable volume measurements compared to the gold standard of manual tracing, while it is considerably faster and more user-friendly to measure liver volumes as well as liver growth in PLD.

## Supplementary information

ESM 1 (DOCX 1529 kb)

## Abbreviations

ADPKD: Autosomal dominant polycystic kidney disease
ADPLD: Autosomal dominant polycystic liver disease
CT: Computed tomography
HU: Hounsfield units
LoA: Limits of agreement
MMWP: Multimodality Workplace
MRI: Magnetic resonance imaging
PLD: Polycystic liver disease
TLV: Total liver volume

## Declarations

### Guarantor

The scientific guarantor of this publication is Joost P.H. Drenth.

### Statistics and biometry

One of the authors (Maatje D.A. van Gastel) has significant statistical expertise regarding the analyses in our manuscript.

### Study subjects or cohorts overlap

Some study subjects or cohorts have been previously reported in Radboudumc and UMC Groningen studies with PLD patients. The current cohort however has not been reported earlier because patients could only be included if two axial CT scans, at least 6 months apart, were available.

### Methodology

• cross-sectional study

• multicenter study
