# Supplementary material for: Validation of a semi-automatic method to measure total liver volumes in polycystic liver disease on computed tomography — high speed and accuracy
Source: Eur Radiol. 2023 Jan 14;33(5):3222–31. doi: 10.1007/s00330-022-09346-6 (PMC10121488; doi:10.1007/s00330-022-09346-6)
Supplement: Supplementary file 1 — (DOCX 1529 kb) [file 330_2022_9346_MOESM1_ESM.docx]

**SUPPLEMENTAL DATA**

**
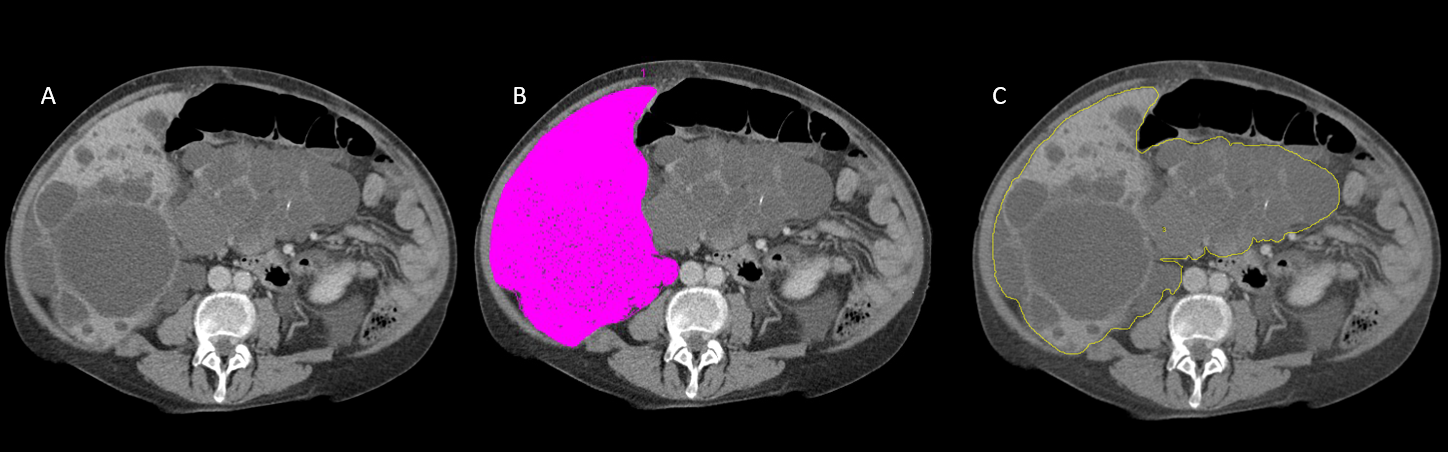

Supplemental Figure 1. Outlier in the comparison of manual and semi-automatic liver growth.** Semi-automatically, TLV on CT 1 was 11626 ml and CT 2 was 11617 ml. Manually, TLV on CT 1 was 11510 ml and on CT 2 was 13211 ml. Segmentation data showed that at the manual measurement of CT 2, the right kidney was assumed to be liver (panel C) while the boundary between right kidney and liver was set properly during the semi-automatic measurement (panel B).


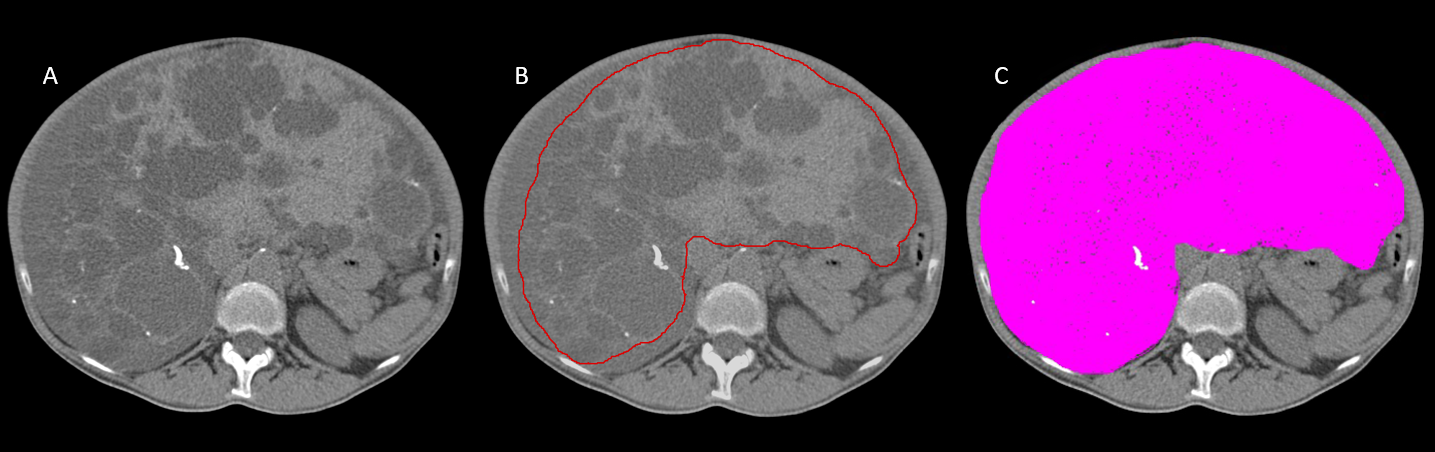
***Supplemental figure 2. Image of the segmentation tools.*** *A = unsegmented slice, B = manual segmentation with Pinnacle, C = Semi-automatic segmentation*

|  | **Manual TLV ml** | **Semi-automatic TLV ml** | **P-value** |
| --- | --- | --- | --- |
| n=10 | 4124.8  [2230.0-5956.9] | 4189.3  [2356.1-6065.4] | 0.114 |
|  | **Manual TLV ml** | **Syngo.Via TLV ml** | **P-value** |
| n=10 | 4124.8  [2230.0-5956.9] | 4265.8  [2301.2-6215.0] | 0.007 |

**Supplemental Table 1. Comparison of manual versus semi-automatic and manual versus Syngo.Via measurements.** TLV = total liver volume. Comparison between liver volumes with Wilcoxon signed rank tests

**
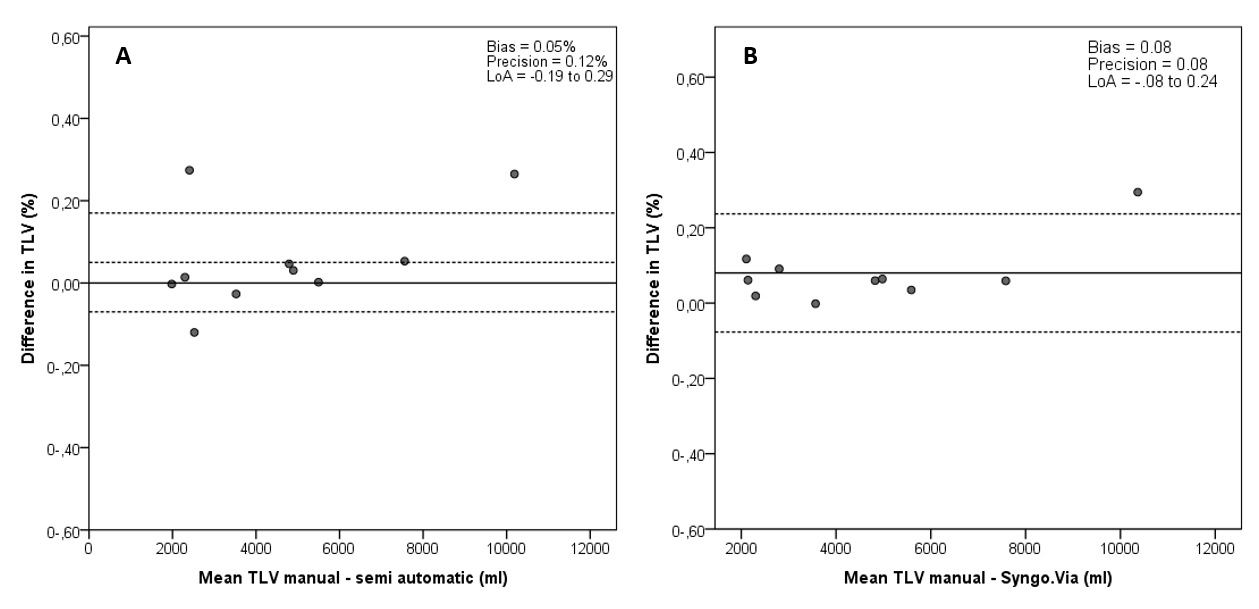
**

**Supplemental Figure 2. Bland Altman plots for cross sectional TLV measurements compared between manual versus semi-automatic (panel A) and manual versus Syngo.Via measurements (panel B).** TLV = total liver volume, LoA = Limits of agreement

|  | **Manual** | **Syngo.Via** | **p-value manual vs Syngo.Via** |
| --- | --- | --- | --- |
| **Inter-reader Coefficient Variation**, % | 1.21% | 1.01% | 0.445 |
| **Intra-reader Coefficient Variation**, % | 0.63% | 0.54% | 0.333 |
| **Measurement Time**, hour:minutes:seconds [IQR] | 0:50:23  [0:46:42 – 1:18:15] | 0:48:19  [0:34:17 – 0:56:03] | 0.917 |

**Supplemental Table 2. Inter- and intra-reader variability for manual and Syngo.Via measurements.** Comparison for manual versus Syngo.Via measurements was made using Wilcoxon signed rank tests


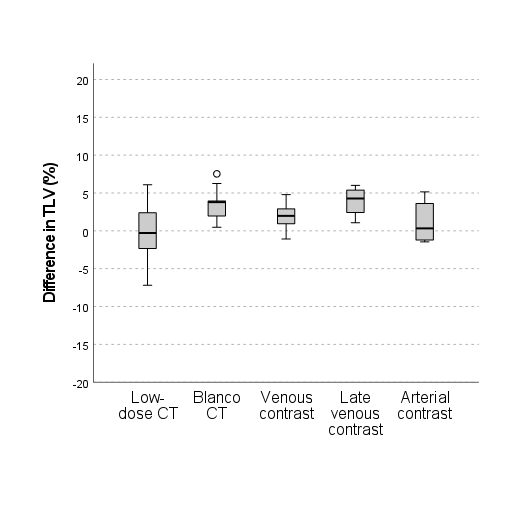

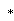


**Supplemental Figure 3. Subgroup analysis of the comparison of manual and semi-automatic measurements between different types of contrast**. On the y-axis, the difference in TLV (%) between the manual and semi-automatic measurements is given, compared to the mean of both. The only groups that differ significantly, are the low-dose CT-scans versus the blanco CT-scans (p=0.04, Kruskal Willis). Between blanco CT-scans and CT-scan with either venous, late venous or arterial contrast, there were no differences.
